# Frequency and clinicopathological features of somatic neoplasms arising in ovarian mature teratomas: a single-center experience

**DOI:** 10.3389/pore.2026.1612455

**Published:** 2026-07-10

**Authors:** Şenay Yıldırım, Gül Alkan Bülbül, Döndü Nergiz, Hülya Tosun Yıldırım, Arif Hakan Önder, Işın Üreyen

**Affiliations:** 1 Department of Pathology, Antalya Training and Research Hospital, Antalya, Türkiye; 2 Department of Perinatology, Antalya Training and Research Hospital, Antalya, Türkiye; 3 Department of Pathology, Health Sciences University, Antalya Training and Research Hospital, Antalya, Türkiye; 4 Department of Medical Oncology, Antalya Training and Research Hospital, Antalya, Türkiye; 5 Department of Gynecological Oncology, Health Sciences University, Antalya Training and Research Hospital, Antalya, Türkiye

**Keywords:** malignant transformation, mature teratoma, ovary, somatic neoplasm, squamous cell carcinoma, adult-type granulosa cell tumor, glioblastoma

## Abstract

**Objective:**

Somatic neoplasms arising in mature teratomas (SN-MT) are rare clinical entities that pose significant diagnostic and therapeutic challenges. This study aims to determine the frequency of SN-MT and evaluate the clinicopathological characteristics that distinguish these cases from uncomplicated mature teratomas (MT).

**Methods:**

We retrospectively reviewed 500 cases of presumed ovarian mature teratomas evaluated at a single center between 2006 and 2021. After strict exclusion criteria, 385 patients were included. All histopathological slides underwent systematic pathological re-evaluation by expert pathologists. Clinical, radiological, and laboratory data were compared between the MT and SN-MT groups.

**Results:**

The overall incidence of somatic neoplasms (benign and malignant) was 3.1% (n = 12), with a malignant transformation rate of 2.07% (n = 8). The histological spectrum was highly heterogeneous and comprised both benign and malignant entities. Benign tumors identified in MTs included paraganglioma, choroid plexus papilloma, and adnexal tumor, whereas malignant tumors included squamous cell carcinoma (SCC), glioblastoma, anaplastic astrocytoma, adult-type granulosa cell tumor, and mixed carcinoma. The SN-MT group exhibited significantly larger mean tumor diameters compared to the MT group (9.45 ± 4.22 cm vs. 6.67 ± 3.32 cm, p < 0.05). On gross examination, mixed solid–cystic architecture was a powerful predictor of neoplasia (83.3% in SN-MT vs. 22.3% in MT, p < 0.0001). Preoperative serum tumor markers (CA 125, CA 19-9, and CEA) showed no significant discriminatory value. False-negative findings on intraoperative frozen section analysis may be attributable to the focal distribution of these lesions.

**Conclusion:**

SN-MT is a rare but histologically diverse condition. Large tumor size and the presence of solid components on macroscopic examination should raise suspicion of somatic transformation. Given the limitations of frozen section analysis and serum markers, rigorous macroscopic sampling and expert pathological examination remain the gold standard for diagnosis. While early-stage disease carries a favorable prognosis, the heterogeneity of subtypes necessitates individualized management.

## Introduction

Mature teratomas (MT) are common benign germ cell tumors composed of well-differentiated tissues from three embryonic layers [1]. Ovarian MTs, particularly the mature cystic subtype (MCT), represent 95% of all teratomas and 10%–20% of all ovarian neoplasms [2]. While typically benign, these tumors can rarely give rise to secondary somatic malignancies, posing significant clinical and prognostic challenges.

Somatic neoplasms arising in mature teratomas (SN-MT), often termed “somatic malignant transformation,” occur in 1%–2% of cases [3]. Despite their rarity, increased risk is associated with advanced patient age, larger tumor size, and prolonged duration of the tumor. While the majority of somatic neoplasms that develop from mature teratomas are malignant, benign or borderline neoplasms have also been documented on occasion. Squamous cell carcinoma (SCC) is the most frequently reported subtype. Other entities include adenocarcinoma, sarcomas (particularly rhabdomyosarcoma), neuroendocrine tumors, melanoma, and rare hematologic malignancies [4].

The pathogenesis of SN-MT remains unclear, with proposed mechanisms including chronic inflammation and genetic instability. Molecular studies suggest roles for TP53 and PIK3CA alterations, though it remains uncertain whether these events originate from benign tissue or secondary malignant clones [5, 6]. Clinically, SN-MT typically presents with nonspecific symptoms like abdominal pain or pelvic masses. While serum markers like SCC antigen and CEA may be elevated in specific cases, they offer limited diagnostic utility [7, 8]. Consequently, preoperative differentiation remains difficult, necessitating histopathological confirmation.

Malignant transformation in mature teratomas is characterized by the presence of invasive malignancy within a background of benign teratomatous tissue [9, 10]. Malignant areas often demonstrate stromal invasion, desmoplastic reactions, marked cytologic atypia, increased mitotic activity, and necrosis. Because these lesions are frequently focal and heterogeneously distributed, small malignant foci may be missed during routine sampling. Therefore, extensive and systematic gross sampling, particularly of the solid areas, mural thickening, necrosis, and hemorrhage, is essential. Immunohistochemistry is critical for distinguishing malignancies arising in mature teratomas from primary or metastatic ovarian malignancies [11–13].

Prognosis depends on histologic subtype, stage, and surgical success, with radical surgery serving as the cornerstone of therapy [14, 15]. Adjuvant treatments should be individualized based on the somatic component rather than conventional germ cell regimens. Given that current evidence is largely limited to small series, the true frequency and prognostic determinants of SN-MT remain poorly defined [16, 17]. This study aims to determine the frequency of SN-MT and compare clinicopathological features across various histologic subtypes.

## Materials and methods

### Study design and case selection

**FIGURE 1:**
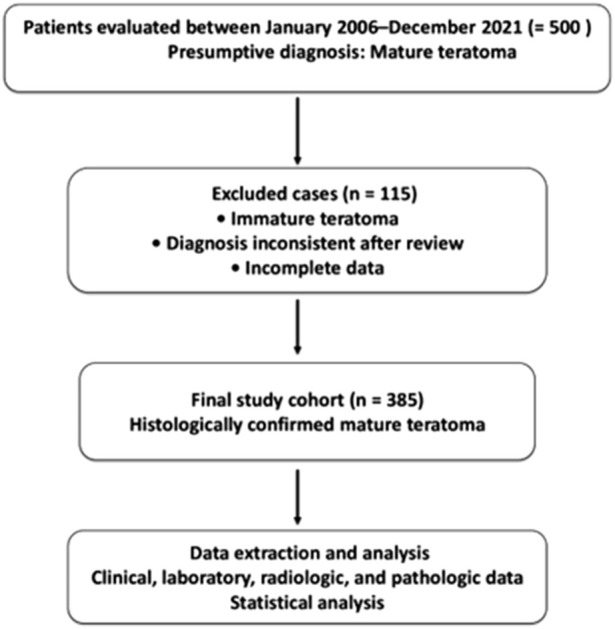
Flow diagram illustrating patient selection and formation of the final study cohort.

This retrospective study included cases evaluated in our pathology department between January 2006 and December 2021, with a diagnosis or presumptive diagnosis of mature teratoma. A total of 500 female patients were screened. Cases were excluded if (i) the final histopathologic diagnosis was immature teratoma; (ii) the diagnosis was not consistent with mature teratoma upon review; or (iii) the clinical, radiologic, or pathologic data were incomplete. After applying the exclusion criteria, 115 patients were excluded, and 385 women with mature teratomas were included in the final analysis (Figure 1).

### Data collection

Clinical, laboratory, and pathological data were retrospectively obtained from patient records and hospital information systems. Clinical variables included age, tumor location (right/left ovary), surgical procedures performed, and treatments received. Laboratory variables included preoperative serum CA 125, CA 19-9, and CEA levels when available.

### Histopathological evaluation

Pathological variables assessed included tumor size, sectional characteristics (cystic, solid, or mixed cystic), and accompanying macroscopic findings. For histopathological examination, systematic sampling was performed, ensuring that at least one tissue section was obtained for every centimeter of the tumor’s maximum diameter. Tissue samples were fixed in 10% neutral buffered formalin for 24–48 h, routinely processed, and embedded in paraffin blocks. Sections with a thickness of 3–4 μm were cut and stained with hematoxylin and eosin (H&E). To ensure diagnostic accuracy, all histopathological slides were re-evaluated by the authors based on current diagnostic criteria to confirm the final diagnosis.

### Immunohistochemistry

In cases where the development of a secondary neoplasm within a mature teratoma was suspected, immunohistochemical (IHC) staining (P40, P63, GFAP, S100, Calretinin, Chromogranin, Synaptophysin, KI 67, etc.) was performed for definitive tumor typing. For IHC analysis, 3-4 μm thick sections were taken from representative paraffin-embedded tumor blocks and placed on poly-L-lysine-coated slides. Immunohistochemical staining was performed using the Roche Ventana Benchmark Ultra system, following the manufacturer’s instructions.

### Statistical analysis

Descriptive statistics are presented as frequency and percentage for categorical variables and as mean ± standard deviation, median, minimum–maximum, and 25th (Q1) and 75th (Q3) percentiles for continuous variables. For categorical comparisons, Pearson’s chi-square test was used when <20% of the expected cell counts were <5; otherwise, Fisher’s exact test was used. Normality was assessed using the Shapiro–Wilk test. As the continuous variables did not follow a normal distribution, group comparisons were performed using the Mann–Whitney U test. All analyses were conducted using IBM SPSS Statistics version 23.0, and statistical significance was set at p < 0.05.

### Ethics approval

This study was reviewed and approved by the ethics committee of Antalya Training and Research Hospital (approval number: 11/3, Date: 23.7.2020). All procedures performed in studies involving human participants were in accordance with the ethical standards of the institutional and/or national research committee and with the 1964 Helsinki declaration and its later amendments or comparable ethical standards.

## Results

A total of 385 women with histologically confirmed mature teratomas were included. The incidence of benign and malignant somatic neoplasms originating from mature teratomas was found to be 3.1% (n = 12). The frequency of malignant transformation from mature teratomas was found to be 2.07% (n = 8). The histological spectrum was heterogeneous, comprising both benign and malignant entities. Benign tumors identified in MTs included paraganglioma (16.6%), choroid plexus papilloma (8.3%), and adnexal tumor (8.3%), while malignant tumors included squamous cell carcinoma (SCC) (33.3%), glioblastoma (8.3%), anaplastic astrocytoma (8.3%), adult-type granulosa cell tumor (8.3%), and mixed carcinoma (8.3%).

Among the 373 MT cases (96.9%) without somatic neoplasia, the mean age was 37.75 ± 14.18 years (range, 13–83 years). The tumors were located in the right (48.2%), left (45.6%), and bilateral (6.2%) ovaries. The mean tumor diameter was 6.67 ± 3.32 cm (range, 1.5–17). On gross examination, 77.7% were cystic, and 22.3% were solid–cystic.

The mean age of the SN-MT group was 43.0 ± 18.27 years (range, 20–73 years). Tumors were located in the right ovary in 75% of the cases and in the left ovary in 25% of the cases. It was observed that none of the SN-MT cases was bilateral. The mean tumor diameter was 9.45 ± 4.22 cm (range, 3.5–15). Grossly, 83.3% of the SN-MT cases showed mixed solid–cystic components, whereas only two cases (16.7%) were purely cystic (Table 1).

**TABLE 1:** Comparison of age and tumor size between MT and SN-MT groups.

Diagnostic group	MT	MT	SN-MT	SN-MT	*p*
Diagnostic group	Mean ± SD (Min–Max)	Median (Q1–Q3)	Mean ± SD (Min–Max)	Median (Q1–Q3)	​
Age (years)	37.76 ± 14.19 (13–82)	36 (27–47)	43.00 ± 18.28 (20–73)	37 (28.5–60.5)	0.390
Left-sided tumor size (cm)	6.76 ± 3.78 (0.8–35)	6 (4.5–8)	14.0 ± 1.0 (13–15)	14 (13–15)	0.006
Right-sided tumor size (cm)	6.36 ± 2.74 (0.1–20)	6 (4.5–8)	7.94 ± 3.74 (3.5–14)	7 (5–10)	0.243

MT: Mature teratoma SN-MT: somatic neoplasms arising in mature teratomas.

The Mann Whitney U test was used. Q1: 25. Percentile and Q3:75. Percentile.

There was no significant difference between the MT and SN-MT groups in terms of age (p = 0.390) or the proportion of patients aged <45 years versus ≥45 years (p = 0.333). Tumor laterality (right/left/bilateral) did not differ significantly between the groups (p = 0.170). When tumor size was analyzed by laterality, left-sided tumors differed significantly between the groups (p = 0.006), with larger tumors in the SN-MT group; right-sided tumors did not differ significantly (p = 0.243). Gross cut-surface characteristics differed markedly between the groups (p < 0.0001). MTs were predominantly cystic, whereas SN-MTs were predominantly mixed solid–cystic (Table 2).

**TABLE 2:** Comparison of clinical and gross features between MT and SN-MT groups.

Variable	MT n (%)	SN-MT n (%)	Total n (%)	*p*	Odds ratio* (95% CI)
Age (years)					
<45	267 (71.6)	7 (58.3)	274 (71.1)	0.333^2^	1.845 (0.573–5.943)
≥45	106 (28.4)	5 (41.7)	111 (28.9)	​	​
Total	373 (100)	12 (100)	385 (100)	​	​
Gross cut-surface					
Cystic	290 (77.7)	2 (16.7)	292 (75.5)	<0.0001^2^	17.169 (3.689–79.904)
Solid + cystic	83 (22.3)	10 (83.3)	93 (24.5)	​	​
Total	373 (100)	12 (100)	385 (100)	​	​
Tumor laterality					
Right	180 (48.2)	9 (75.0)	189 (49.1)	0.170^1^	​
Left	170 (45.6)	3 (25.0)	173 (45.0)	​	​
Bilateral	23 (6.2)	0 (0)	23 (5.9)	​	​
Total	373 (100)	12 (100)	385 (100)	​	​

1 Pearson chi-square test and2: Fisher’s Exact test were used. *: Odds ratio is obtained from 2x2 tables.

Preoperative serum CA 125 was available for 274 patients, CA 19-9 for 251, and CEA for 191 patients, respectively. No significant differences were observed between the MT and SN-MT groups for CA 125, CA 19-9, or CEA (all p = 0.99). The marker levels are summarized in Table 3.

**TABLE 3:** Comparison of serum tumor marker levels between MT and SN-MT groups.

Variable	MT n (%)	SN-MT n (%)	Total n (%)	*p*	Odds ratio (95% CI)
CA 125 (U/mL)					
<35	233 (87.9)	8 (88.9)	241 (88.0)	0.99	0.91 (0.11–7.518)
≥35	32 (12.1)	1 (11.1)	33 (12.0)	​	​
Total	265 (100)	9 (100)	274 (100)	​	​
CA 19-9 (U/mL)					
<37	182 (74.9)	6 (75.0)	188 (74.9)	0.99	0.995 (0.196–5.058)
≥37	61 (25.1)	2 (25.0)	63 (25.1)	​	​
Total	243 (100)	8 (100)	251 (100)	​	​
CEA (ng/mL)					
<2.5	156 (85.2)	7 (87.5)	163 (85.3)	0.99	0.825 (0.098–6.979)
≥2.5	27 (14.8)	1 (12.5)	28 (14.7)	​	​
Total	183 (100)	8 (100)	191 (100)	​	​

Intraoperative frozen section evaluation was performed in 7/12 SN-MT cases (58.3%). Frozen sections were reported as benign in 4 cases and malignant in 3 cases. Among those reported as malignant, the final diagnoses included SCC (n = 2) and mixed carcinoma (n = 1). Among the frozen section–benign cases, final pathology revealed glioblastoma, anaplastic astrocytoma, and SCC, indicating false-negative frozen section results. In five SN-MT cases where intraoperative frozen sections were not performed, microscopic evaluation of permanent sections revealed the development of choroid plexus papilloma, paraganglioma, skin adnexal tumor, and adult-type granulosa cell tumor. Intraoperative frozen section results, individual surgical and medical treatment plans for 12 SN-MT patients are reported in Table 4.

**TABLE 4:** Presentation of somatic neoplasms arising in mature teratomas.

Case	Age	Type of procedure	Result of frozen	Immunohistochemistry	Diagnosis	Complementary surgery	Stage	Chemotherapy status	Follow-up (months)	Status
1	60	TAH + BSO + bilateral pelvic lymphadenectomy+ Omentectomy	Malign	P63 (+) Kİ 67:%60	SCC	Not performed	IC	Not received	108	Deceased
2	33	Bilateral cystectomy	Benign	P63 (+) Kİ 67:%80	SCC	Unilateral salpingo-oophorectomy + omentectomy + Paraortic and bilateral pelvic lymphadenectomy	IA	Not received	50	Alive
3	73	TAH + BSO + Paraortic and bilateral pelvic lymphadenectomy+ Omentectomy	Malign	P63 (+)	SCC	Not performed	IA	Not received	77	Deceased
4	39	TAH + BSO + Paraortic and bilateral pelvic lymphadenectomy + Omentectomy	Not performed	P63 (+) Kİ67:%70	SCC	Not performed	IIIB	1st line: Carboplatin + Paclitaxel 2nd line: Oxaliplatin + Gemcitabin	132	Deceased
5	61	TAH + BSO + Paraortic and bilateral pelvic lymphadenectomy+ Omentectomy	Malign	SCC: P40 (+) Mucinous adenocarcinoma: CK20(+) MUC1(+) MUC2(+) High-grade adenocarcinoma: p53 overexpression	Mixed carcinoma	Not performed	IIIA2	VAC-IE (Vincristine, Adriamycin (Doxorubicin) and Cyclophosphamide (VAC) + Ifosfamide and etoposide	67	Deceased
6	20	Left ovarian cystectomy	Benign	GFAP (+) ATRX: Retained IDH-1: Wild type Kİ67:%30	Glioblastoma	Left USO + omentectomy	IIIA1	BEP (bleomycin + etoposide + Cisplatin)	46	Alive
7	22	Left ovarian cystectomy	Benign	GFAP (+) ATRX: Nuclear expression lost IDH-1: Mutant Kİ67:%5	Anaplastic astrocytoma	Left USO + omentectomy + Paraortic and bilateral pelvic lymphadenectomy	IA	BEP (bleomycin + etoposide + Cisplatin)	79	Alive
8	50	Right ovarian cystectomy	Not performed	İnhibin (+) Calretinin (+)	Adult type granulosa cell tumor	TAH + BSO	IA	Not received	126	Alive
9	35	Right ovarian cystectomy	Not performed	S100 (+) GFAP (+) Kİ67:%1	Choroid plexus papilloma	Not performed	​	Not received	107	Alive
10	31	Right ovarian cystectomy + appendectomy	Not performed	Chromogranin (+) Synaptophysin (+) CD56 (+) Kİ67:%2	Paraganglioma	Not performed	​	Not received	50	Alive
11	26	Bilateral cystectomy	Not performed	Chromogranin (+) Synaptophysin (+) CD56 (+) Kİ67:%1	Paraganglioma	Not performed	​	Not received	55	Alive
12	66	TAH + BSO	Benign	CK7(+)ER (+) PR (+)	Benign skin appendage tumor	Not performed	​	Not received	108	Alive

TAH + BSO: Transabdominal hysterectomy + Bilateral salpingo-oophorectomyUSO: Unilateral salpingo-oophorectomySCC: squamous cell carcinoma.

**FIGURE 2:**
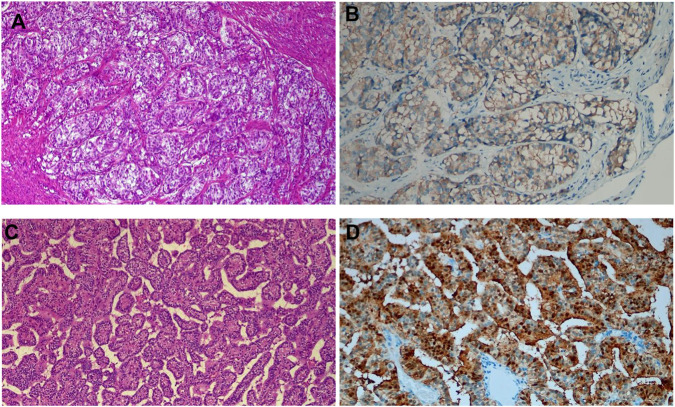
Benign neoplasms arising in mature teratomas **(A)** Paraganglioma with characteristic “Zellballen” pattern (H&E, X100). **(B)** Moderate cytoplasmic synaptophysin expression in paraganglioma cells (Synaptophysin, X200). **(C)** Choroid plexus papilloma with complex papillary architecture (H&E, X200). **(D)** S100 protein expression in the epithelial lining of choroid plexus papilloma (S100, X200).

Among the benign neoplasms arising within mature teratomas, paraganglioma was characterized by the classic “Zellballen” nesting pattern of tumor cells separated by a delicate fibrovascular stroma (Figure 2A); immunohistochemically, these cells demonstrated moderate cytoplasmic synaptophysin expression (Figure 2B). The choroid plexus papilloma cases exhibited a complex papillary architecture lined by uniform epithelial cells (Figure 2C), which showed strong S100 protein positivity along the epithelial lining (Figure 2D). Additionally, adnexal skin appendage tumors were identified by their well-differentiated follicular or sebaceous structures closely associated with the ectodermal components of the teratoma.

**FIGURE 3:**
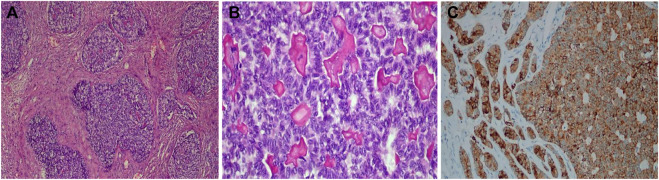
Adult-type granulosa cell tumor arising within a mature teratoma. **(A)** The tumor exhibits granulosa cells arranged in trabecular and insular patterns, separated by fibromatous stroma (H&E, X100). **(B)** Microfollicular pattern with numerous Call-Exner bodies. The neoplastic cells are cuboidal-to-polygonal with scant cytoplasm and pale, uniform, angulated, and grooved (coffee-bean) nuclei (H&E, X400). **(C)** Diffuse cytoplasmic positivity for inhibin within the tumor cells (Inhibin, X100).

Within the malignant category, the tumors followed diverse lineages. SCC displayed sheets and nests of atypical squamous cells infiltrating the stroma, prominent keratin pearl formation, and extensive intercellular bridges, alongside diffuse nuclear p63 positivity. Adult-type granulosa cell tumors showed prominent trabecular, insular, and microfollicular growth patterns (Figure 3A), the latter containing numerous pathognomonic Call-Exner bodies; the neoplastic cells presented cuboidal-to-polygonal morphology with scant cytoplasm and characteristic pale, angulated, and grooved nuclei (Figure 3B), accompanied by diffuse cytoplasmic positivity for inhibin (Figure 3C) and calretinin.

**FIGURE 4:**
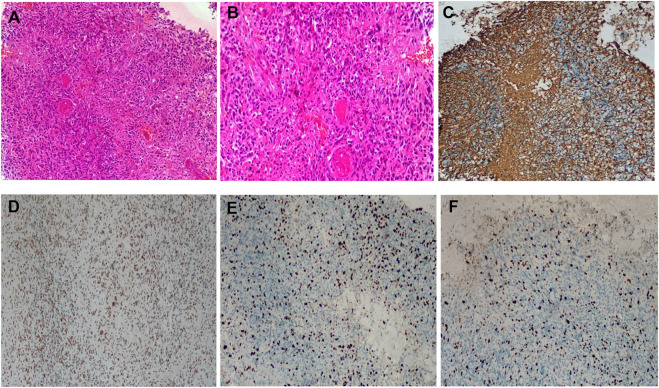
Glioblastoma arising within a mature teratoma. **(A)** The tumor exhibits markedly increased cellularity, prominent nuclear atypia, and areas of necrosis (H&E, X100). **(B)** Pseudopalisading necrosis with neoplastic cells surrounding a central necrotic zone and frequent atypical mitotic figures (H&E, X200). **(C)** Diffuse GFAP positivity in tumor cells (X200). **(D)** Retained nuclear ATRX expression within the neoplastic cells (X100). **(E)** Diffuse and strong nuclear p53 overexpression in tumor cells (X100). **(F)** High Ki-67 proliferation index within the tumor cells (X100).

**FIGURE 5:**
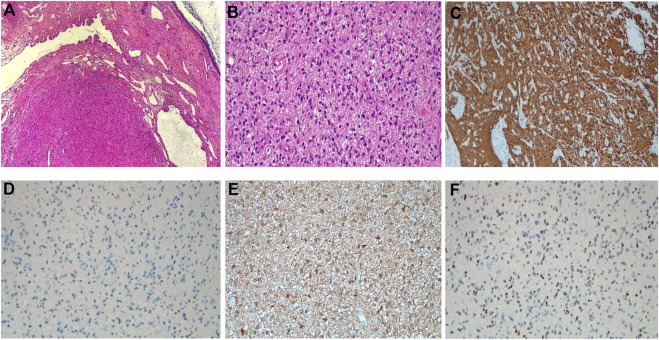
Anaplastic astrocytoma arising within a mature teratoma. **(A)** Glial cell proliferation adjacent to the epithelial components of the mature teratoma (H&E, X40). **(B)** Tumor cells demonstrating moderate pleomorphism, in the absence of necrosis and marked proliferative activity (H&E, X200). **(C)** Diffuse GFAP positivity in tumor cells (X100). **(D)** Loss of nuclear ATRX expression in neoplastic cells (X200). **(E)** Strong immunoreactivity for mutant IDH-1 (IDH1-R132H) within tumor cells (X200). **(F)** Low Ki-67 proliferation index, quantified at approximately 5% (X200).

Among the neuroepithelial malignancies, glioblastoma displayed high-grade features, including markedly increased cellularity, prominent nuclear atypia, frequent atypical mitoses, and areas of necrosis with pseudopalisading (Figures 4A,B) Immunohistochemically, the tumor cells showed diffuse GFAP positivity (Figure 4C), retained nuclear ATRX expression (Figure 4D), strong nuclear p53 overexpression (Figure 4E), and a remarkably high Ki-67 proliferation index (Figure 4F). In contrast, anaplastic astrocytoma presented as a glial proliferation adjacent to mature teratoma epithelia, exhibiting moderate pleomorphism without necrosis or prominent proliferative activity (Figures 5A,B). Structurally, these cells revealed diffuse GFAP positivity (Figure 5C), loss of nuclear ATRX (Figure 5D), strong mutant IDH-1 (IDH1-R132H) immunoreactivity (Figure 5E), and a low Ki-67 index (5%) (Figure 5F).

**FIGURE 6:**
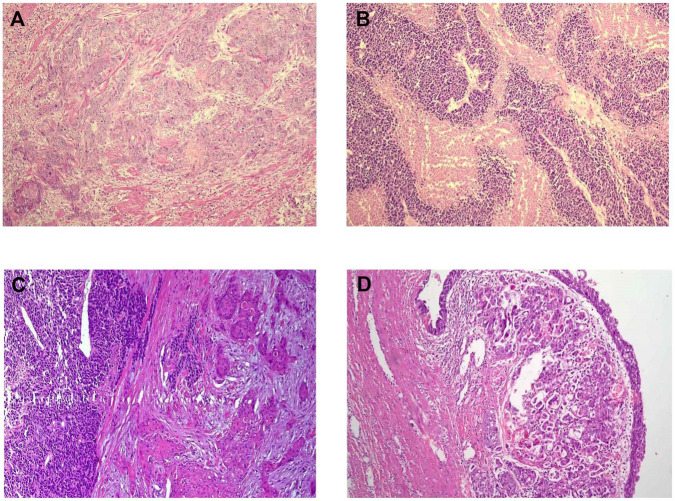
Histopathological components of the mixed carcinoma arising in a mature cystic teratoma. **(A)** Areas of squamous cell carcinoma (SCC) showing invasive nests of malignant epithelial cells (H&E, X100). **(B)** Foci of high-grade adenocarcinoma characterized by complex architectural patterns within extensive areas of necrosis (H&E, X100). **(C)** The transition zone demonstrating the junction between the SCC and high-grade adenocarcinoma components (H&E, X100). **(D)** Mucinous adenocarcinoma component composed of cells with large vacuolated cytoplasm, exhibiting a pattern of single-cell infiltration within the stroma (H&E, X100).

The mixed carcinoma case comprised three distinct malignant components. The first component consisted of infiltrative nests of atypical keratinized and non-keratinized squamous cells showing p40 positivity (Figure 6A). Adjacent to these areas, a high-grade adenocarcinoma component was identified in a necrotic background, characterized by pleomorphic cells with papillary architecture and p53 overexpression (Figure 6B). The intimate juxtaposition and transition between the squamous cell carcinoma and high-grade adenocarcinoma components were clearly demonstrated (Figure 6C). Finally, a small focus of mucinous adenocarcinoma was present, composed of pleomorphic cells with prominent nucleoli and abundant vacuolated cytoplasm, showing positivity for MUC1 and MUC2 immunostains (Figure 6D). Notably, p53 demonstrated a wild-type expression pattern in both the squamous and mucinous adenocarcinoma. components. In the MT group, cystectomy was performed in 60.5% of the patients, USO in 20%, and TAH + BSO in 19.5%. The SN-MT group underwent Total Abdominal Hysterectomy + Bilateral Salpingo-Oophorectomy (TAH + BSO) in 41.7% of the patients, unilateral salpingo-oophorectomy (USO) in 41.7%, and cystectomy in 16.6%. Of the eight cases exhibiting malignant transformation, four were diagnosed following TAH + BSO, while the remaining four were diagnosed via unilateral/bilateral ovarian cystectomy. According to the 8th edition of the American Joint Committee on Cancer (AJCC) staging system, the distribution was as follows: Stage I (n = 5) and Stage III (n = 3). The clinicopathological and surgical characteristics of the SN-MT cases are detailed in Table 4.

At diagnosis, para-aortic lymph node metastasis was detected only in the mixed carcinoma case; at 31 months, the patient developed right adrenal metastasis. The adrenal metastasis was confirmed to be of ovarian origin, as whole-body PET-CT imaging demonstrated no other primary tumour focus, thereby excluding a synchronous or metachronous secondary malignancy. The SN-MT group was followed for a mean of 80.3 ± 6.13 months (range, 50-132).

## Discussion

In the present study, we evaluated the frequency and clinicopathological characteristics of somatic neoplasms arising in mature teratomas in a large cohort over an extended study period. The incidence of malignant transformation was 2.07%, which falls within the reported range of 0.17%–5.5% in the literature, although it is higher than the 0.54% rate reported by Neville et al. [18]. This difference may be related to the long study period, systematic archival review, and detailed re-evaluation of surgical specimens, which likely improved detection sensitivity, particularly for rare histological variants and focal lesions.

The histological spectrum in our cohort was heterogeneous, comprising both benign and malignant entities. Squamous cell carcinoma (SCC) was the most frequent subtype, accounting for approximately one-third of cases, consistent with previous studies [18–20]. This predominance is biologically plausible given the frequent squamous differentiation within mature teratomas. Rare entities, including paraganglioma, adult-type granulosa cell tumor, and central nervous system-type tumors, further highlight the broad differentiation potential and histological diversity of these lesions.

From a histogenetic perspective, mature teratomas (MTs) and adult granulosa cell tumors (AGCTs) arise from distinct embryological lineages [21]. Their coexistence in the same ovary is rare, with fewer than 15 cases reported [22, 23] The most widely accepted explanation is the collision tumor theory, although alternative hypotheses, including divergent differentiation, have also been proposed [21]. Molecular studies, particularly FOXL2 mutation analysis, support the independent origin of AGCTs [24, 25]. In our case, the synchronous presentation of AGCT and MT in the right ovary of a 50-year-old patient represents a rare entity. Morphologically, a 2.2 cm solid area located within the wall of a 5 cm MT exhibited classic AGCT features, characterized by uniform cells displaying microfollicular/insular patterns and nuclear grooves (coffee-bean nuclei); this diagnosis was confirmed by diffuse immunohistochemical positivity for inhibin and calretinin. Although molecular analysis for FOXL2 mutation could not be performed due to institutional limitations, the distinct histological demarcation between the two components, the absence of a transitional differentiation zone, and their entirely different embryological lineages rule out the possibility of a mixed/combined tumor. These findings strongly support that our case represents a pathological model of a “collision tumor,” characterized by the coincidental coexistence of two independent neoplasms.

The occurrence of glial neoplasms within mature cystic teratomas is exceptionally rare and is considered within the spectrum of monodermal teratomas. Although neuroectodermal differentiation is relatively common in mature teratomas, malignant transformation into high-grade astrocytic tumors is extremely uncommon. Clinically, these aggressive secondary malignancies typically affect young women, presenting primarily with non-specific abdominal pain and signs of mass effect [26]. From a diagnostic and macro-morphological perspective, evaluating these specimens carries inherent difficulties; because the astrocytic component arises directly within the background of an ovarian germ cell tumor framework, precisely measuring or isolating the exact boundaries and size of the astrocytoma can often be highly challenging [27]. This underscores the critical importance of extensive sampling and a comprehensive IHC panel to prevent diagnostic pitfalls. In our glioblastoma case, the diagnosis was supported by diffuse GFAP positivity, ATRX retention, p53 overexpression, a high Ki-67 proliferation index, and IDH1 (R132H) negativity, consistent with an IDH-wildtype phenotype. However, given the limited number of reported cases, the molecular profile of ovarian glioblastomas remains incompletely defined.

While some studies have suggested advanced age as a risk factor for somatic malignant transformation, others have failed to demonstrate it as an independent predictor [28]. In the present analysis, no significant differences were observed in age when assessed as a continuous variable or when stratified by a 45-year threshold. However, the broader age distribution observed in the SN-MT group indicates a need for more in-depth clinical and pathological evaluation of older patients.

Tumor size and macroscopic features appear to be important indicators of somatic neoplastic transformation [29]. In our study, SN-MTs were significantly larger and more frequently demonstrated mixed solid–cystic architecture compared with MTs. The presence of solid areas, mural nodules, or necrosis should therefore raise suspicion for malignant transformation and prompt extensive sampling.

No statistically significant difference was observed between the MT and SN-MT groups in terms of tumor location. Although right-sided tumors were significantly more frequent in the SN-MT group, this finding should be interpreted with extreme caution due to the limited sample size of our cohort. This distribution reflects a random variation specific to small sample sizes rather than a clear biological mechanism. Similarly, the absence of somatic neoplasia in bilateral mature teratomas in our cohort should not be generalized and is likely due to the limited number of cases.

Serum tumor markers (CA 125, CA 19-9, and CEA) showed no significant difference between groups, consistent with previous reports indicating limited diagnostic value in predicting somatic transformation [30].

Although intraoperative frozen section analysis was performed in only a limited number of cases (n = 7) in our cohort, our descriptive observations highlight the inherent diagnostic challenges associated with these lesions. In our small subset, the presence of focal and heterogeneous malignant components appeared to contribute to the difficulty of intraoperative assessment. While no definitive statistical conclusions can be drawn due to this sample size limitation, our experience suggests that careful procurement of samples, specifically targeting the solid components of the mass during intraoperative frozen section, may help minimize diagnostic pitfalls. Likewise, during gross evaluation of mature teratomas, meticulous and comprehensive sampling of heterogeneous areas remains a critical practice to facilitate the identification of potential SN-MT areas.

Malignant transformation was identified in eight patients, four of whom were diagnosed after TAH + BSO and four after USO. The preference for more radical surgical approaches in SN-MT cases reflects the impact of suspicion of malignancy on surgical decision-making [31]. Despite the favourable outcomes observed in the majority of cases, particularly in patients with early-stage and completely resected tumors, advanced-stage patients demonstrated a need for chemotherapy.

Finally, several methodological limitations are acknowledged. The retrospective design inherently limits the data completeness and uniformity of measurements. An incomplete assessment of serum tumor markers may reduce the statistical power. The relatively small number of SN-MT cases constrained detailed subtype-specific analyses and generalizability. The lack of molecular and genetic investigations has limited mechanistic and prognostic interpretations. Nevertheless, the systematic pathological re-evaluation of all cases by expert pathologists, the long follow-up period, and the large overall cohort strengthened the significance of our findings. Future multicenter prospective studies incorporating molecular profiling are warranted to elucidate the biology of somatic neoplasms that arise in mature teratomas.

In conclusion, this study demonstrated that somatic neoplasms arising in mature teratomas represent a rare but highly heterogeneous clinicopathological entity. Larger tumor size and presence of solid components are the most reliable features associated with somatic neoplastic transformation. Our findings contribute to increased diagnostic awareness among pathologists and clinicians, and provide practical insights for the early recognition of this uncommon but clinically significant phenomenon. Further large-scale, multicenter, prospective studies are essential to validate these observations and facilitate the development of standardized diagnostic and therapeutic algorithms.

## Data Availability

The original contributions presented in the study are included in the article/supplementary material, further inquiries can be directed to the corresponding author.
